# Cascaded deep transfer learning on thoracic CT in COVID-19 patients treated with steroids

**DOI:** 10.1117/1.JMI.8.S1.014501

**Published:** 2020-12-09

**Authors:** Jordan D. Fuhrman, Jun Chen, Zegang Dong, Fleming Y. M. Lure, Zhe Luo, Maryellen L. Giger

**Affiliations:** a The University of Chicago, Committee on Medical Physics, Department of Radiology, Chicago, United States; b Renmin Hospital of Wuhan University, Department of Radiology, Wuhan, China; c MS Technologies Corp., Rockville, Maryland, United States; d Fudan University, Zhongshan Hospital, Department of Critical Care Medicine, Shanghai, China; e Fudan University, Zhongshan Hospital, Department of Critical Care Medicine, Xiamen Branch, Xiamen, China

**Keywords:** coronavirus disease-19, transfer learning, methylprednisolone, deep learning, computed tomography

## Abstract

**Purpose:** Given the recent COVID-19 pandemic and its stress on global medical resources, presented here is the development of a machine intelligent method for thoracic computed tomography (CT) to inform management of patients on steroid treatment.

**Approach:** Transfer learning has demonstrated strong performance when applied to medical imaging, particularly when only limited data are available. A cascaded transfer learning approach extracted quantitative features from thoracic CT sections using a fine-tuned VGG19 network. The extracted slice features were axially pooled to provide a CT-scan-level representation of thoracic characteristics and a support vector machine was trained to distinguish between patients who required steroid administration and those who did not, with performance evaluated through receiver operating characteristic (ROC) curve analysis. Least-squares fitting was used to assess temporal trends using the transfer learning approach, providing a preliminary method for monitoring disease progression.

**Results:** In the task of identifying patients who should receive steroid treatments, this approach yielded an area under the ROC curve of 0.85±0.10 and demonstrated significant separation between patients who received steroids and those who did not. Furthermore, temporal trend analysis of the prediction score matched expected progression during hospitalization for both groups, with separation at early timepoints prior to convergence near the end of the duration of hospitalization.

**Conclusions:** The proposed cascade deep learning method has strong clinical potential for informing clinical decision-making and monitoring patient treatment.

## 1 Introduction

The recent outbreak of the 2019 coronavirus has disrupted the global economy, exhausted medical resources, and adversely affected millions of individuals.^1^^–^^3^ The associated disease (COVID-19) typically manifests through pulmonary dysfunction, including development of acute respiratory distress syndrome through COVID-19 pneumonia.^4^ Because an effective therapeutic drug is yet to be approved, steroid administration has been widely implemented by clinicians to treat severe cases of COVID-19 despite the many side effects that have been recognized.^5^^–^^7^ In particular, methylprednisolone is a common steroid used for COVID-19 treatment due to its demonstrated impact in treating inflammatory symptoms in other respiratory infections.^8^^,^^9^ However, patient reaction to steroid administration is variable, depending on many factors including patient age, smoking history, and other comorbidities. Thoracic imaging through chest computed tomography (CT) is used clinically to aid in differential diagnoses, monitor disease progression/severity, and, in the case of steroid administration, inform treatment regimen, which is especially critical for COVID-19 due to the significant burden that this disease places on medical resources. Deep transfer learning methods may have a role in identifying the amount and type of medical resources that will be needed throughout patient hospitalization.

### 1.1 COVID-19 Presentation in CT Scans

The primary finding of COVID-19 patients on CT scans is peripheral and nodular/mass-like ground-glass opacities (GGO), typically presenting bilaterally with a predominance for lower lung lobes.^10^^,^^11^ As the disease progresses to a severe state, GGO is observed more centrally, with infiltration and consolidation.^10^ The visualization of COVID-19 through CT is strongly dependent on the amount of time between virus contraction and scan acquisition, causing inaccurate diagnosis for radiologist reading.^12^ However, CT has been used by clinicians as the most effective way to visualize the progress of treatment for many pulmonary diseases, including lung cancer and pneumonia, by assessing changes in diseased tissue size, shape, and density. However, these evaluations are often qualitative and subjective, leading to inconsistent judgments and detrimental consequences in patient care. Exploring quantitative metrics such as volume and density has shown improved evaluation accuracy; however, these measurements depend on accurate, consistent delineation of the diseased tissue, which requires consensus from radiologists to draw, reconcile, and prioritize their delineation. Deep learning has the potential to overcome these difficulties and provide quantitative assessments of disease progression.

### 1.2 Deep Learning for CT Scan Assessment

In the past decade, machine learning, including deep learning, techniques have shown outstanding potential in a variety of detection, diagnosis, and prognosis evaluations, providing improvements in both performance and consistency.^13^^,^^14^ Specifically, convolutional neural networks (CNN) can be optimized to perform a specific task by training parameters using a subset of available data.^15^^,^^16^ However, there is often a lack of sufficient medical imaging data to successfully train an optimized CNN.^15^ One potential strategy to overcome this limitation is transfer learning, which optimizes a network to perform one task, then directly transfers network weights for application to some other related task.^15^^–^^19^ For example, a network trained to classify between images of cats and dogs can then be transferred with minimal additional training for detection of lung nodules. Note that while this allows for the extraction of higher-order features that would be otherwise unavailable, there is no guarantee that the transferred network will be successful for the new task. To account for this, network parameters can be fine-tuned for the new task using part of the new dataset.^20^ In this way, a transfer learning technique can be developed that takes advantage of deep networks while achieving improved optimization for the new task. This study utilizes such a strategy, with a cascaded transfer learning technique for prognostic and temporal evaluations of CT scans obtained from COVID-19 patients.

## 2 Methods

### 2.1 Database

In this study, 41 patients each confirmed to have COVID-19 and each with multiple CT scans acquired at different timepoints were retrospectively analyzed with demographic information and imaging parameters as summarized in Table 1. Throughout the course of treatment, each patient progressed to a severe disease stage, requiring a variety of treatments including ventilators, antivirals, and steroids. In particular, the decision to administer steroids was reached based on a combination of symptom severity and CT abnormalities, with 27 of the 41 patients determined by an expert intensivist as severe enough to necessitate steroid administration.

**Table 1:** Database information.

	Pretreatment analysis	During treatment analysis
Number of cases	41 total scans	221 total scans (41 cases)
		3 scans (3 cases)
		4 scans (7 cases)
Number of CT scans acquired (N)	NA	5 scans (10 cases)
		6 scans (14 cases)
		7 scans (6 cases)
		8 scans (1 case)
Average number of timepoints (SD)	NA	Mean (5.39) SD (1.21)
Dates of acquisition	February 1, 2020 to March 30, 2020	
Sex	Male (19) and female (22)	
Age	Mean (63.8); SD (11.5); range (40-87)	
Scanner manufacturer	GE Medical Systems	
kVp	120	
Pitch	Range (0.9844 to 1.750)	
Slice thickness	0.625 mm (211 scans) and 5 mm (10 scans)	

### 2.2 Pretreatment Cascaded Transfer Learning of CT for COVID-19 Management Recommendation

**Fig. 1:**
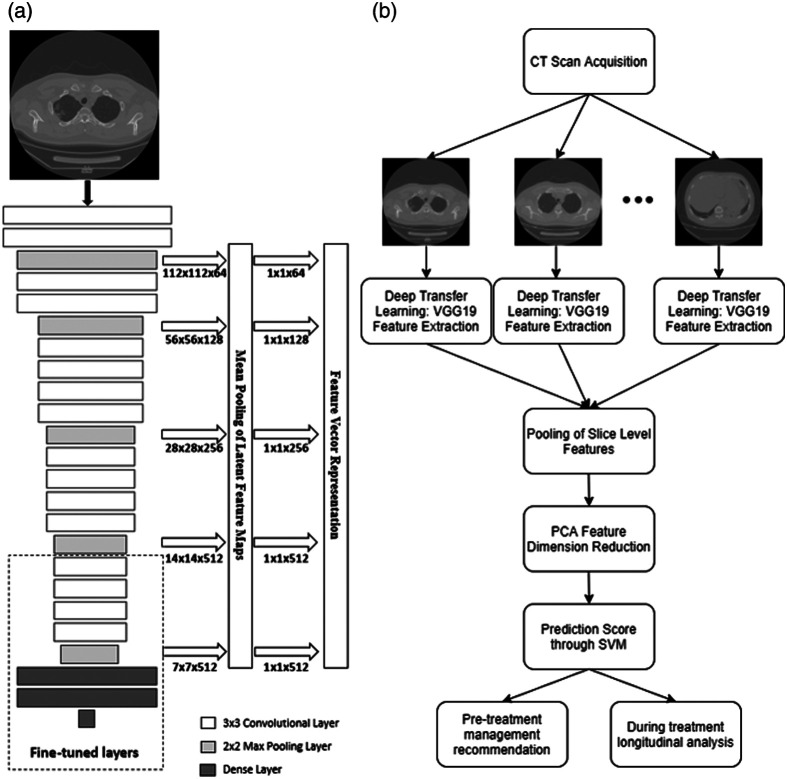
(a) Schematic of the pretrained VGG19 network feature extraction approach operating on a two-dimensional (2D) CT section. Max pooling layer features with the given dimensions were averaged and concatenated to produce a representative feature vector for each slice. (b) Full cascaded transfer learning workflow for pretreatment assessment and during-treatment monitoring analysis. The feature extraction scheme displayed in (a) is utilized at the “Deep Transfer Learning: VGG19 Feature Extraction” stage of (b).

The VGG19 architecture, a technique from the ImageNet competition, is commonly used in transfer learning through pretraining on a collection of millions of natural images.^21^ In a prior study, fine tuning of the ImageNet-trained VGG19 network had been conducted in the task emphysema detection.^22^ This fine-tuned VGG19 network was directly applied to CT sections from the COVID-19 cases in this study and features were extracted for transfer learning using a technique similar to that described by Antropova et al. in which information is taken from the max-pooling layers of the architecture [Fig. 1(a)].^19^ These extracted features were then averaged in the axial scanning direction (e.g., individual slice features combined to form a CT-scan-level representation) and principal component analysis (PCA) was performed on the extracted COVID-19 scan features to reduce to 39 quantitative features per scan.^23^ Final classification was then conducted on the features using a linear support vector machine (SVM).^24^

Due to the limited size of this database, a leave-one-out scheme was used to train the SVM for the classification between cases that required steroid administration and those that did not.^25^ To attain a prognostic evaluation of COVID-19 patients, only the initial CT scan obtained for each patient was evaluated, at which point the patients presented with varying degrees of disease severity. The leave-one-out evaluation approach used 40 cases for SVM training and 1 case for testing; this was repeated 41 times so that each case belonged to the testing set exactly once. Over the 41 iterations, the SVM produced an output “prediction score” related to the likelihood of requiring steroid treatment for each case. The prediction score yields an estimate of the likelihood that a patient would require steroids for treatment based on their CT scan (higher prediction score indicates higher likelihood of recommendation for steroid treatment).

The classification performance was evaluated using receiver operating characteristic (ROC) curve analysis on the prediction scores by comparison with the actual treatment as had been clinically determined by an expert intensivist.^26^ The area under the ROC curve (AUC) served as the figure of merit in this analysis. The full workflow of the cascaded transfer learning technique is shown in Fig. 1.

### 2.3 During Treatment Transfer Learning for COVID-19 Longitudinal Analysis of CT Scans

In addition to predicting which patients should require steroids, it is also important to monitor disease progression throughout hospitalization. Thus, the cascaded transfer learning technique was also applied to the CT scans obtained at all timepoints; the only difference between this during-treatment assessment technique and the pretreatment assessment technique in Sec. 2.2 is that the linear SVM classifier for during-treatment assessment was trained using features from all longitudinally acquired CT scans, not only the initial scan of each case.

After each of the CT scans within a case underwent deep transfer learning feature extraction and subequent PCA feature reduction, the SVM was trained again using the leave-one-out-by-case paradigm based on receiving steroids or not with incorporation of all timepoints in both training and testing. Thus, each patient received a set of model prediction scores, one prediction score for each timepoint’s CT scan. The time of steroid administration was eliminated as a confounding factor by adjusting the ground truth for a given scan based on whether or not steroids were utilized at any point after that scan’s acquisition. For example, a case with 5 timepoints could have a timepoint ground truth of 1 at timepoints 1, 2, and 3 and ground truth of 0 at timepoints 4 and 5, indicating no steroids were administered after the fourth scan acquisition. This is represented as a set {1,1,1,0,0}. The SVM prediction score at a midtreatment timepoint can then be interpreted as a prediction of whether the patient will undergo steroid treatment at any point after that midtreatment scan. Note that during the leave-one-out process, all CT scans of a given case were held out from training when it was used for testing.

The assessment of temporal changes throughout hospitalization was performed through least squares fitting. All patients within the study cohort began with moderate severity and advanced to a more serious condition, followed by recovery and subsequent hospital discharge. This was observed for cases who were treated with steroids and those who were not. Thus, the least squares technique was used to fit second-order polynomials, which, to some degree, match the expected progression for both groups.

## 3 Results

**Fig. 2:**
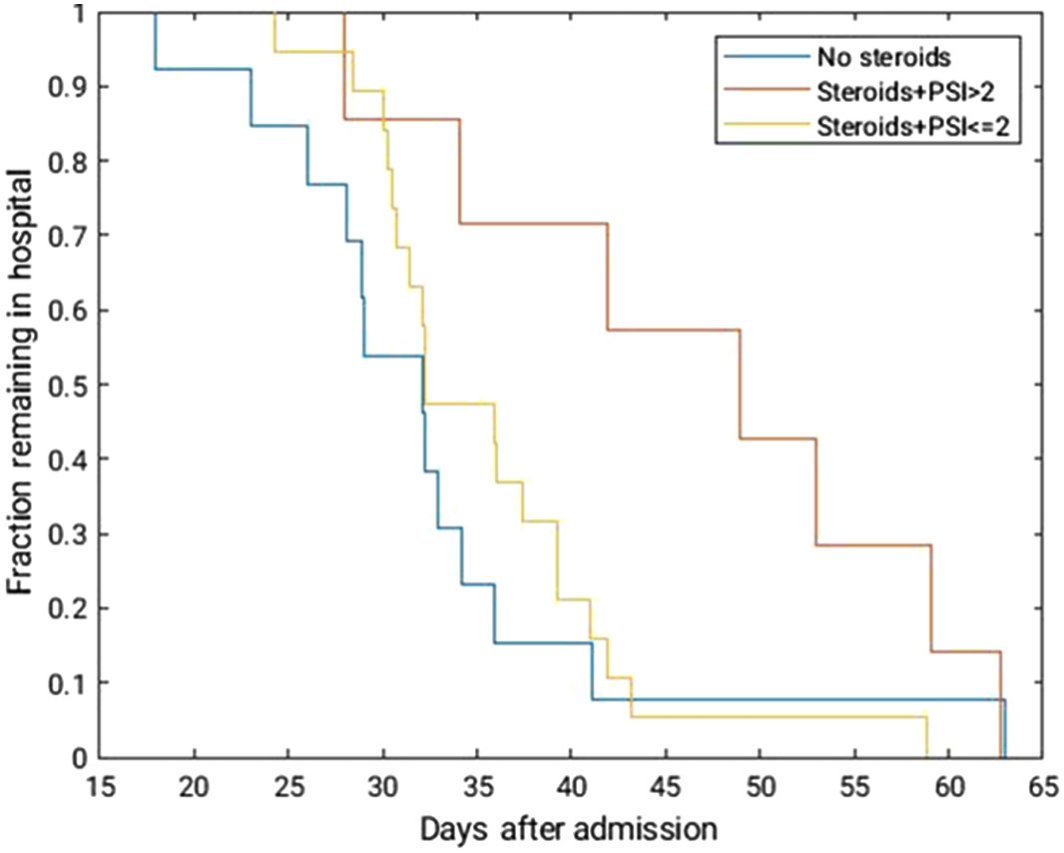
Kaplan–Meier survival analysis assessing the duration of hospitalization with changing treatment and initial PSI score. In general, patients who received steroid treatments were hospitalized for longer periods of time, with particularly long stays for patients with more severe initial symptoms. This is expected, as more severe cases require increased treatment and recovery time.

For our database, to demonstrate that the medical resources needed to adequately treat patients who need steroids and those who do not are notably different, Kaplan–Meier survivor analysis was performed with time of hospitalization exchanged for time of survival (Fig. 2).^27^ In particular, patients who demonstrated a higher pneumonia severity index (PSI) grade experienced much longer hospitalization times than those with a lower PSI.^28^ This demonstrates the need for appropriate, consistent management, and treatment of patients who have progressed to severe disease stages, especially during peak resurgences of COVID-19 when a heavy burden is placed on medical systems to replenish and maintain resources.^29^^,^^30^

### 3.1 Pretreatment Cascaded Transfer Learning of CT for COVID-19 Management Recommendation

**Fig. 3:**
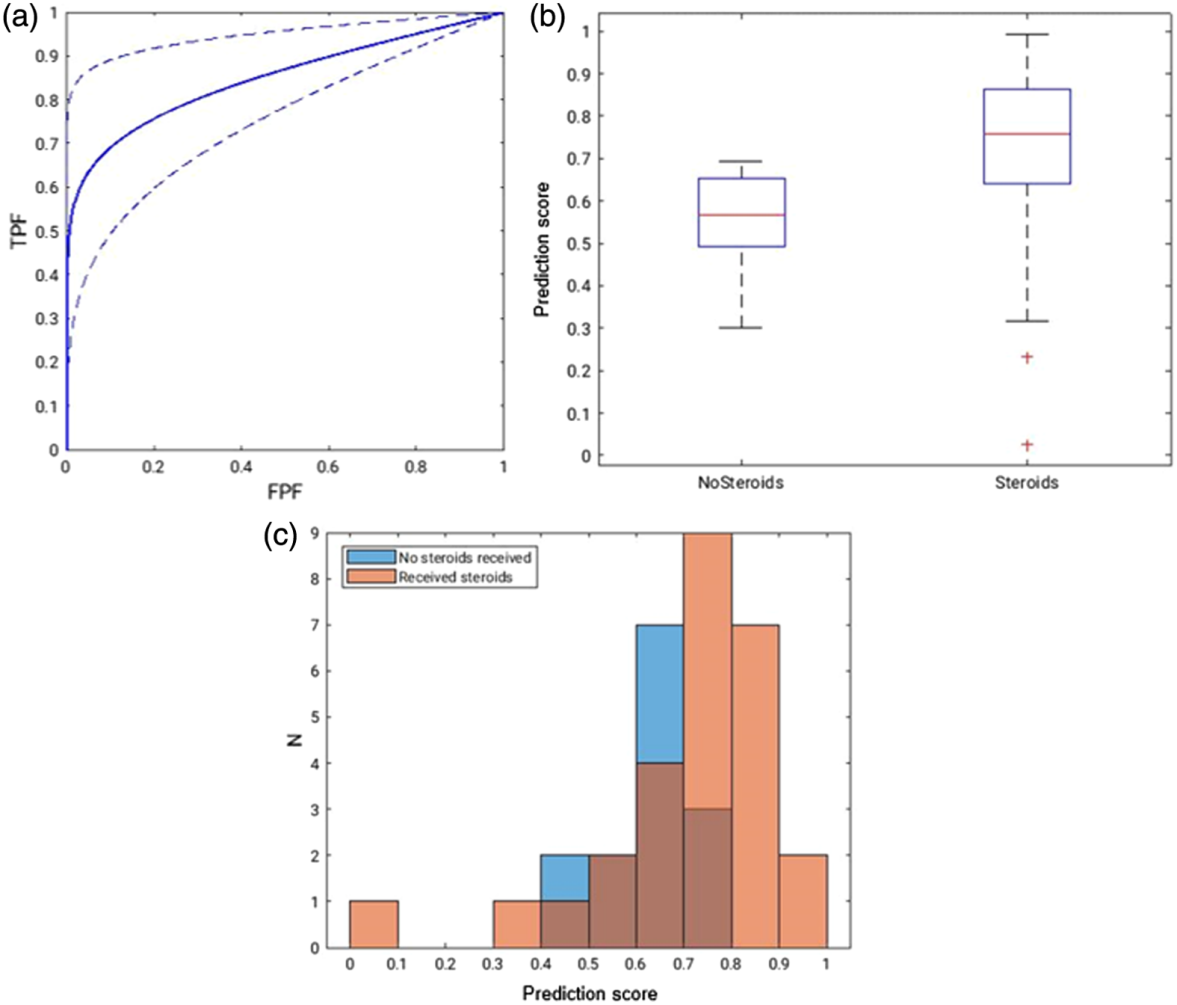
(a) The ROC curve demonstrating the classification ability of the cascade transfer learning method for estimating the likelihood that a COVID-19 patient would be recommended for steroid treatment or not. AUC=0.85±0.10 with the accompanying 95% TPF confidence interval. (b) Distribution of deep learning scores of those patients who received steroids and those who did not. Note, this was obtained only based on the initial CT scan. Based on this plot, the method suggests steroid administration more frequently than the experienced intensivist (using a cutoff of 0.5). The red lines denote the median scores, the blue boxes include 50% of scores, while the black whiskers include all scores within 2σ of the mean. (c) Further demonstration of the separation/overlap of the deep learning score between the two classes.

In the predictive analysis of the initial (pretreatment) CT scans, the cascaded transfer learning technique produced an AUC of 0.85±0.10 based on proper binormal ROC analysis in the task of distinguishing between cases that were recommended for steroid administration and those that were not, demonstrating a statistically significant improvement in comparison to a random chance AUC of 0.5 (p=0.002) [Fig. 3(a)].^31^ By analyzing the distribution of the deep learning scores based on the true steroid administration [Figs. 3(b) and 3(c)], there were two outliers within the distribution of cases that received steroid treatments. These two outliers belonged to patients with low PSI scores and who were young compared to the mean population age (ages 41 and 48).

### 3.2 During-Treatment Transfer Learning for COVID-19 Longitudinal Analysis of CT Scans

**Fig. 4:**
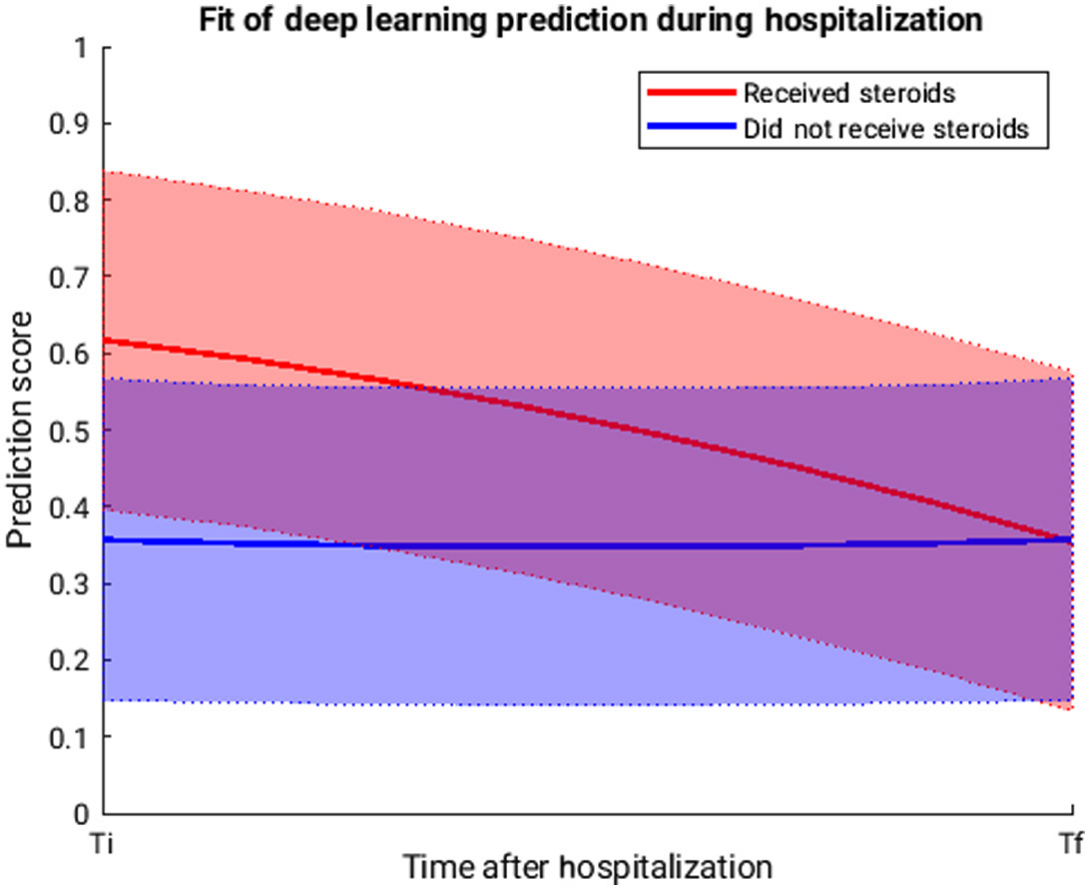
The SVM-output prediction score assessed temporally through least squares fits. The x axis indicates the full duration of hospitalization, with Ti referring to the time of initial CT acquisition and Tf referring to the time of final CT acquisition, which generally occurred shortly before discharge. The shaded regions denote one standard deviation above and below the fit line. Intuitively, this figure follows the example training case discussed in Sec. 2.3, which had early timepoints after which steroids were utilized and late acquisitions after which no steroids were administered.

Preliminary longitudinal analysis was completed through least squares fitting of the raw data (Fig. 4). Due to the variable initial disease state, rate of progression, and treatment schedules, there was significant variation across patients, thus the wide coverage of the shaded regions denoting a one standard deviation range above and below the fit line.

## 4 Discussion

Based on ROC analysis in the task of identifying patients who required steroid treatments and on the longitudinal trends obtained through least squares fitting, the cascaded transfer learning approach showed strong potential for clinical patient management through informing treatment decisions and monitoring patient progression.

While preliminary, this technique demonstrated potential to estimate a likelihood that a patient will progress to a disease stage that is severe enough to necessitate steroid administration during their course of treatment. This holds potential value for allowing hospitals to obtain sufficient medical resources for adequate patient care, including maintenance of steroid supplies, utilization of life saving equipment such as ventilation, extracorporeal membrane oxygenation, and planning for hospital bed occupancy. These are challenges that stressed the medical system during the COVID-19 pandemic, thus this predictive tool could be useful in future resurgences of COVID-19 or other emerging respiratory infectious diseases. Further, this deep transfer learning technique might not only benefit allocation of resources, it could also potentially improve patient management through treatment guidance as indicated by the temporal results matching the treatment decision of the experienced intensivist who provided the ground truth in this study. In particular, this will be useful for regions/clinicians that have little to no experience with COVID-19 (e.g., new faculty, few cases during pandemic), as the cascaded transfer learning technique can aid clinicians in matching previous successful treatments by advocating for or against steroid administration.

Use of deep transfer learning for monitoring treatments may be useful for estimating the amount of resources that will be required throughout patient treatment as demonstrated in our preliminary longitudinal analysis shown in Fig. 4, including hospital beds, ventilators, and medication. The SVM prediction score for this analysis may be interpreted as an expectation of if that patient will require steroids at any point after input CT acquisition. Consequently, if a patient is already experiencing corticosteroid injections for treatment and receives a high prediction score, this suggests that they should continue steroid treatments for some period.

Importantly, the fit lines cannot be used as a direct indicator of disease severity obtained at any individual timepoint during this study; if this were the case, then the fit lines would both demonstrate, on average, a concave mirroring effect showing that the prediction score increases as time progresses, and then decreases as treatment takes effect until a very low level is reached as the disease subsides. This expectation of concave mirroring prediction score is due to the disease progression manifested by radiological findings of COVID-19 in CT scans; with increasing severity, the CT findings exhibit GGOs, more central infiltration, and consolidation, then a return to primarily peripheral GGO as the patient recovers. This mirroring effect is not observed for either classification. The failure to demonstrate a mirroring effect for fit line from steroid administration can be explained by the ground truth used for model training, which was a decision to treat with steroids or not at some point after scan acquisition. While this is inherently related to disease severity, this treatment decision was not solely based on imaging findings; it also considered clinical symptoms and other factors (e.g., age). Thus, the fit line does not directly translate to a temporal assessment of severity during this study.

Instead, the trends should be interpreted as a recommendation for steroid administration. On average, the cases that required steroids demonstrated a larger prediction score for scans acquired upon hospitalization. However, as time after hospitalization increased, the two curves converged, indicating that the recommendation for steroid administration grew weaker over time for those who already received steroids, and demonstrated nearly complete overlap at the termination of hospitalization. This matched expected results because the clinical outcomes of all patients in this cohort were the same, i.e., recovery and subsequent discharge, thus all patients should demonstrate a similar recommendation for steroids upon discharge. This validated the prediction score as a potentially useful clinical measurement.

Clinically, the longitudinal aspect of this study can be used by physicians as a comparison to guide treatment decisions and, in a way, assess treatment response (e.g., is the model recommendation for steroid administration getting stronger or weaker?). Further, consider a patient that has been administered corticosteroids that now produces a CT scan with a low prediction score (e.g., ∼0.3). According to the temporal fits in Fig. 4, this suggests that the patient is likely nearing the end of their hospitalization period and that cessation of steroid administration may be suitable. Alternatively, it is possible that some patients will not follow a progression of predictions scores similar to the fit lines; in this case, the temporal assessments may not be applicable and the clinician should be more reliant on other data (e.g., clinical symptom severity) to determine steroid treatment termination. Thus, the cascaded transfer learning approach in this study demonstrated potential in guiding treatment decisions, monitoring patient progression, and managing medical resources.

### 4.1 Limitations

A key limitation was the lack of cases in which steroid treatment administration was ineffective. In this study, all cases recovered and were subsequently discharged from the hospital. While the responsiveness to these therapies is variable between patients, this was not accounted for in this study, thus inclusion of a larger, more diverse dataset could provide insight into more general application of this technique. This would also further validate the during-treatment analysis which showed wide variance in fit curves.

In addition, the ground truth severity of patients at each imaging timepoint was unavailable, so verification of the least-squares fits was difficult because all classifications in this study were binary. While this preliminary investigation into temporal analysis shows promise, further work toward validating the results must be completed before clinical implementation.

## 5 Conclusions

Due to the significant impact of the COVID-19 pandemic, development of tools for diagnosing disease and improving patient care is critical. In this study, a cascaded transfer learning approach using a fine-tuned VGG19 network in conjunction with PCA and an SVM classifier was able to characterize thoracic CT scans and distinguish between patients who necessitated steroid treatment and those who did not as well as provide a preliminary investigation of automated patient monitoring. Based on the results obtained through ROC analysis and least-squares polynomial fitting, this approach shows the potential of clinical implementation of this technique. With further investigation, this method may guide clinicians to provide more effective treatments and improve patient outcomes.
